# Supplementary material for: Extracellular-matrix-mediated osmotic pressure drives Vibrio cholerae biofilm expansion and cheater exclusion
Source: Nat Commun. 2017 Aug 23;8:327. doi: 10.1038/s41467-017-00401-1 (PMC5569112; doi:10.1038/s41467-017-00401-1)
Supplement: Supplementary file 1 — Supplementary Information [file 41467_2017_401_MOESM1_ESM.pdf]

Description of Supplementary Files

File Name: Supplementary Information
Description: Supplementary Figures, Supplementary Discussion, Supplementary Table and Supplementary References

File Name: Peer Review File

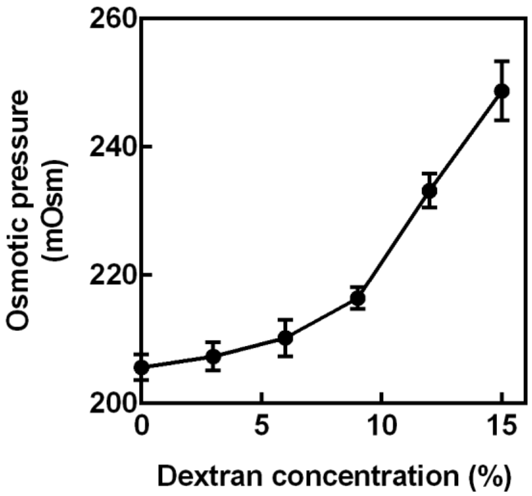

Supplementary Figure 1. Osmotic pressure of M9 medium containing different percentages (w/w) of dextran (~ 2000 kDa) measured by vapor pressure osmometry. Error bars correspond to standard deviations (*n* = 3-5).

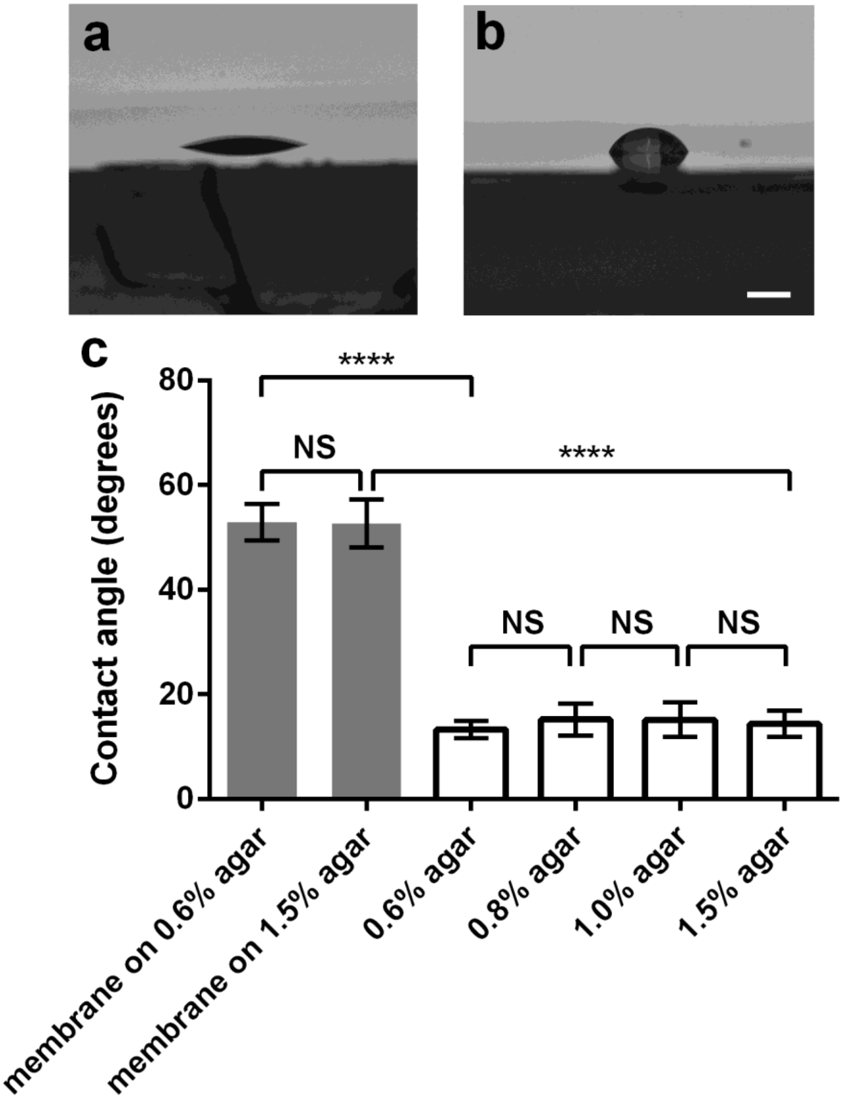

Supplementary Figure 2. Surface contact angle measurements. **a-b**, Representative side-view images of a 1 μL droplet of water on 1.5% agar (**a**) and on a semipermeable membrane atop 1.5% agar (**b**). Scale bar: 0.1 cm. **c,** Contact angles of water on a semipermeable membrane sitting on top of agar (gray bars) and on bare agar (white bars). The contact angle on the semipermeable membrane is significantly larger than that on bare agar. In neither case does the contact angle depend on the agar concentration. All error bars correspond to standard deviations with n = 4.

Unpaired *t*-tests with Welch's correction were performed in **c**. NS denotes not significant; **** denotes $P < 0.0001$.

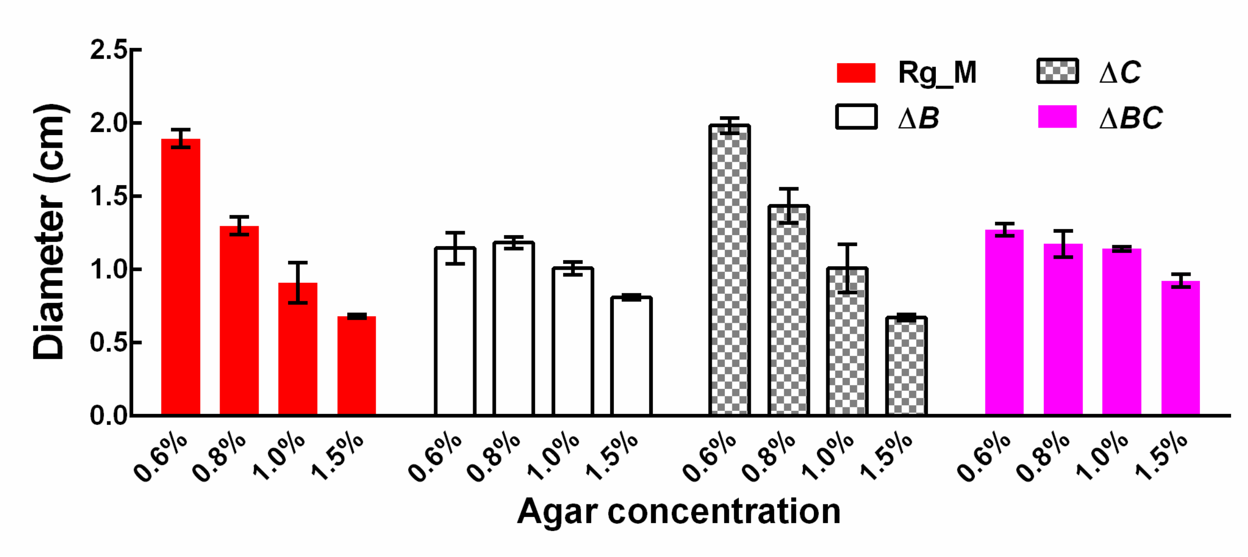

Supplementary Figure 3. Surface adhesion strongly affects the osmotic-pressure-driven expansion of *V. cholerae* colony biofilms. Shown are colony diameters as a function of agar concentration for the Rg_M (red), Δ*bap1* (denoted Δ*B*, white), Δ*rbmC* (denoted Δ*C*, checkerboard), and Δ*bap1*Δ*rbmC* (denoted Δ*BC*, magenta) strains grown for 2 days on LB medium containing the specified percentages of agar. All error bars correspond to standard deviations with $n = 4$.

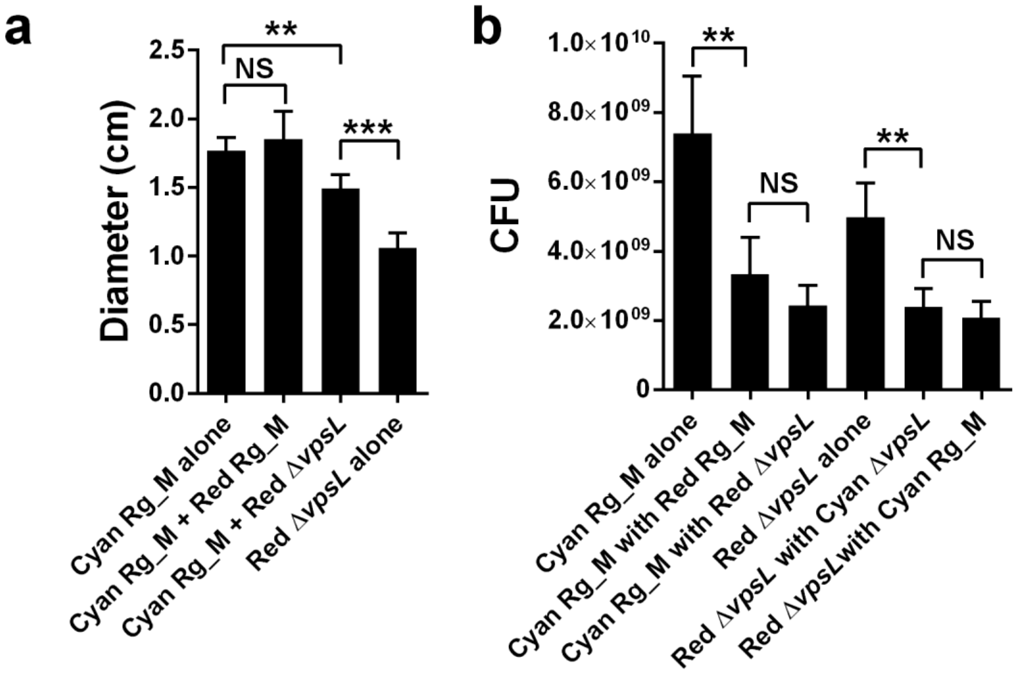

Supplementary Figure 4. Δ*vpsL* cells are cheaters in co-inoculated colony biofilms with the Rg_M strain. **a,** Diameters of 2-day-old colony biofilms grown on LB medium solidified with 0.6% agar when Rg_M cells with the cyan fluorescent marker *mTFP1* are inoculated alone, with an equal number of Rg_M cells labeled with the red fluorescent marker *mKate2*, or with an equal number of Δ*vpsL* cells labeled with *mKate2*. The right-most bar shows the results for the Δ*vpsL* strain labeled with *mKate2* inoculated alone. **b,** Number of live cells for the first strain indicated on the *x*-axis label measured by colony forming units (CFU) when grown alone or in co-inoculated colony biofilms. Unpaired *t*-tests with Welch's correction were performed for all comparisons. All error bars correspond to standard deviations with $n = 5$. Incorporation of Δ*vpsL* cells into the colony biofilm decreased the overall colony biofilm size compared to when the Rg_M strain was grown alone. Incorporation of Δ*vpsL* cells also decreased, albeit modestly, the number of viable cells of the co-inoculated Rg_M strain. Therefore, we conclude that Δ*vpsL* cells

do not contribute to matrix production or to the osmotic-pressure-driven expansion, however they do compete with the co-inoculated Rg_M cells for space and nutrients. NS denotes not significant; ** denotes $P < 0.01$ and *** denotes $P < 0.001$.

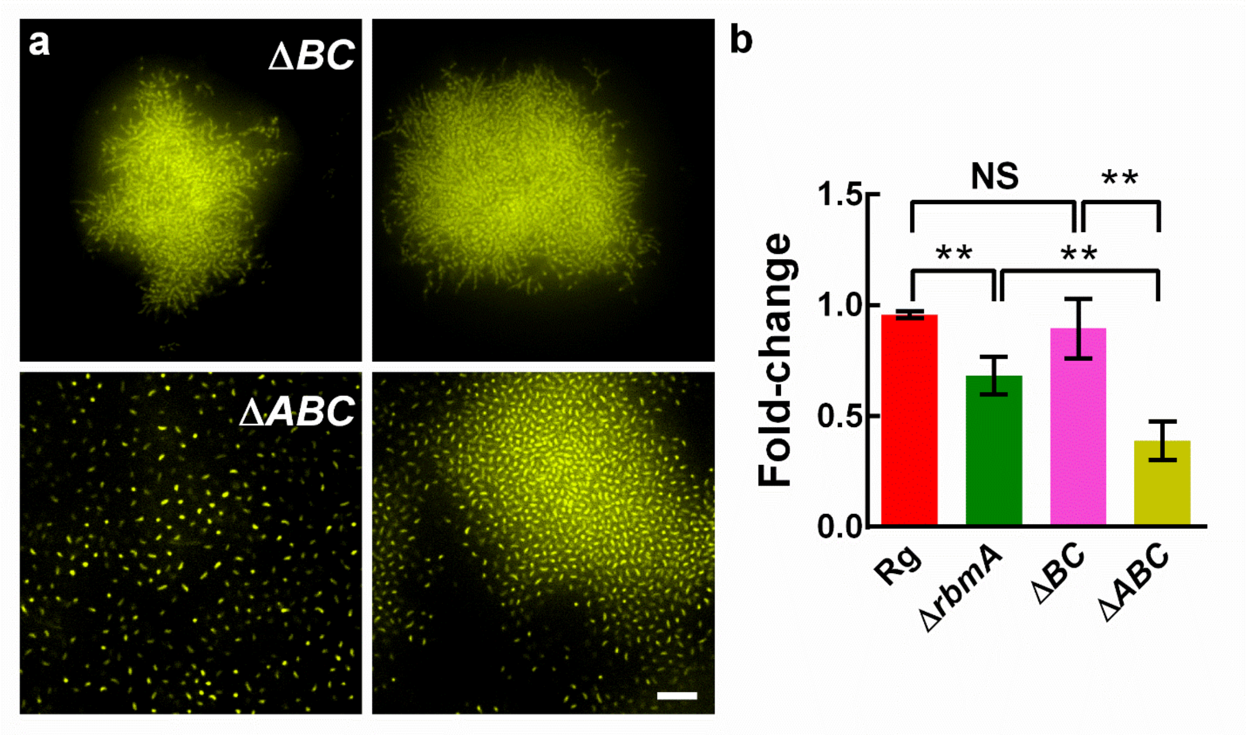

Supplementary Figure 5. Osmotic pressure controls the expansion of submerged *V. cholerae* biofilms **a,** Representative images for submerged biofilms of different *V. cholerae* strains following hyperosmotic shock. Shown are cross-sectional confocal images of submerged Δ*bap1*Δ*rbmC* (*top*) and Δ*rbmA*Δ*bap1*Δ*rbmC* biofilms (*bottom*) before (*left*) and immediately after (*right*) hyperosmotic shock generated by the addition of 15% dextran to the medium. Cells constitutively express *mKO* from the chromosome. Images are at 6 μm above the surface. Scale bar: 10 μm. For these mutant biofilms, it was not possible to identify the original clusters following the medium exchange because the biofilms move during the operation due to lack of surface attachment. Therefore, different clusters are shown before and after the hyperosmotic shock. **b,** Fold-change in volume of submerged biofilms of the designated strains immediately following hyperosmotic shock. Unpaired *t*-tests with Welch's correction were performed in **b**. NS denotes not significant and ** denotes $P < 0.01$. Error bars correspond to standard deviations with $n = 4$.

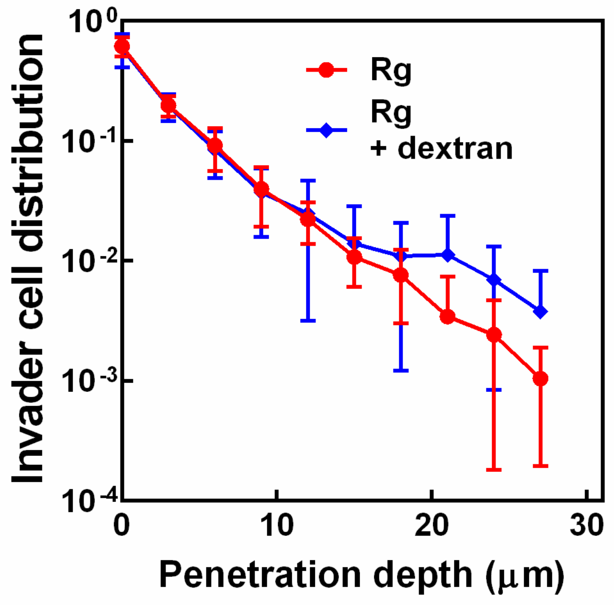

Supplementary Figure 6. Distribution of invader cells in biofilms. Shown is a control experiment for the data provided in Fig. 6d of the main text. Plotted is the invader cell distribution as a function of penetration depth into the resident Rg submerged biofilm without dextran (red circles) and with dextran (blue diamonds). All error bars correspond to standard deviations with $n = 4$. The compactness of submerged Rg biofilms does not change with external osmotic pressure; hence, the susceptibility of Rg biofilms to invasion does not statistically change upon addition of dextran.

Supplementary Discussion

Here, we provide our rationale for the estimation of the internal osmotic pressure in *V. cholerae* submerged biofilms. Previous measurements have shown that, in liquid, EPS producing strains have a 20% lower growth rate than EPS non-producing strains[1]. Hence, we assume that an Rg cell devotes ~ ¼ of its carbon resources to EPS production, assuming carbon to be the limiting energy source and elemental nutrient. The carbon composition of *V. cholerae* EPS[2] is approximately the same as the overall carbon composition of the bacterial cell[3]. Thus, we assume that during the course of doubling, an Rg cell also produces EPS prior to swelling with ¼ the volume of the initial cell. Previous measurements have shown that *V. cholerae* cells are roughly 2 μm long and 1 μm wide in submerged biofilms[4], leading to a rough estimation of cell volume $V_{\text{cell}} \sim 2\ \mu\text{m}^3$. Thus, the unswelled EPS volume is approximately 0.5 μm^3. Once secreted, EPS polymers (with the assistance of matrix proteins) form crosslinks with one another. The matrix material also swells in size due to the osmotic pressure differential between the crosslinked network and the growth medium. To calculate the final swelled volume of the EPS, we assume that EPS polymers homogeneously fill all interstitial spaces between biofilm cells. To calculate the interstitial volume, we first calculate the volume occupied by each cell V'_{cell} in the biofilm by calculating its Voronoi volume[5], which is approximately 5 μm^3 for Rg cells and 14 μm^3 for Δ*rbmA* cells in submerged biofilms. The interstitial volume per cell equals $V'_{\text{cell}} - V_{\text{cell}}$, and from the interstitial volume, we estimate that the EPS material has swelled 6 fold for Rg and 24 fold for Δ*rbmA* submerged biofilms, relative to its nascent, unswelled states. Note, these values are consistent with those generally used in biofilm simulations to estimate the volumes occupied by cells and by EPS polymer[6]. We assume that in the nascent state, the initial polymer volume fraction ϕ_0 is close to the packing fraction of the bacterial cytoplasm, which is around 0.25[7].

Thus, we arrive at $\phi \sim 0.04$ for Rg cells and 0.01 for Δ*rbmA* cells in osmotically swelled submerged biofilms. One can also derive these values by directly estimating the EPS biomass produced by each cell, which yields similar results.

To estimate internal osmotic pressure from ϕ, we need to apply an appropriate scaling law that reflects the conformation of EPS molecules in solution. The *V. cholerae* EPS is a heteropolymer with many α (1-4) carbohydrate linkages[2], rendering it flexible. Measurements show that such flexible polysaccharides assume a conformation close to that of an ideal coil in aqueous solution[8,9]. Therefore, the excluded volume between the sugar units nearly cancels out the hydrogen-bonding-driven intermolecular interactions between water molecules and sugar units[8,9]. With respect to the biofilm environment, the estimated ϕ suggests that the EPS is similar to a semi-dilute polymer solution[10]. Thus, the scaling law $\Pi_{\mathrm{EPS}} \sim kT/b^3 \times \phi^3$ can be employed[10,11], since the leading term $\propto \phi^2$ is zero for an ideal coil. The unit size b used here is close to the Kuhn length measured for flexible polysaccharides such as dextran (0.44 nm)[12], which is approximately the size of one 6-carbon ring.

In the absence of a scaffold of interconnected cells, e.g. in the Δ*rbmA* biofilm, we can extend this calculation by assuming that the swelling of the EPS network due to the osmotic-pressure contrast is balanced by the entropic cost of stretching the polymer strands between the internal crosslinking points in the EPS network. Such classical gel theory[10], in principle, allows one to estimate the number of repeating sugar units N between the crosslinking points via $1/\phi = N^{3/8}/\phi_0^{1/4}$, in which ϕ_0 is the initial polymer volume fraction estimated above. Performing this estimate for the Δ*rbmA* biofilms yields an estimate of $N = 8.6 \times 10^4$. This value seems rather high for the estimated molecular weight of the EPS[13]. Furthermore, we can estimate the correlation

length or mesh size ξ in such a hydrogel. For ideal solutions, $\xi \sim b/\phi$, yields a characteristic length scale of 50 nm. This value is consistent with the fact that beads larger than 50 nm cannot penetrate biofilms whereas smaller beads as well as extracellular enzymes do diffuse into biofilms. However, a strong assumption underlying this calculation is the existence of a homogeneous gel network. We caution that experiments show that *V. cholerae* EPS molecules appear as dense puncta inside biofilms, and they form envelopes around subclusters of cells in Rg biofilms[14]. Hence, applying this general, homogeneous gel theory might be insufficient to accurately capture the detailed biofilm characteristics.

Supplementary Table 1. *E. coli* and *V. cholerae* strains used in this study.

Strains/plasmid	Relevant Features	Reference
E. coli		
S17 λ-*pir*	Wild Type	[15]
V. cholerae		
C6706*str*2	El Tor Wild Type	[16]
JY028	$vpvC^{W240R}$ (denoted Rg)	
JY030	$vpvC^{W240R}$ *lacZ*:P_{tac}-*mKate2*:*lacZ*	[17]
JY031	$vpvC^{W240R}$ *lacZ*:P_{tac}-*mKO*:*lacZ*	[17]
JY040	$vpvC^{W240R}$ Δ*vpsL lacZ*:P_{tac}-*mKate2*:*lacZ*	This study
JY041	$vpvC^{W240R}$ Δ*vpsL lacZ*:P_{tac}-*mKO*:*lacZ*	This study
JY048	$vpvC^{W240R}$ Δ*rbmA lacZ*:P_{tac}-*mKate2*:*lacZ*	[17]
JY049	$vpvC^{W240R}$ Δ*rbmA lacZ*:P_{tac}-*mKO*:*lacZ*	[17]
JY085	$vpvC^{W240R}$ Δ*bap1*Δ*rbmC lacZ*:P_{tac}-*mKO*:*lacZ*	[17]
JY096	$vpvC^{W240R}$ Δ*rbmA*Δ*bap1*Δ*rbmC lacZ*:P_{tac}-*mKO*:*lacZ*	[17]
JY283	$vpvC^{W240R}$ Δ*pomA* (denoted Rg_M)	This study
JY284	$vpvC^{W240R}$ Δ*pomA* Δ*rbmA*	This study
JY285	$vpvC^{W240R}$ Δ*pomA* Δ*bap1*Δ*rbmC*	This study
JY286	$vpvC^{W240R}$ Δ*pomA* Δ*rbmA*Δ*bap1*Δ*rbmC*	This study
JY287	$vpvC^{W240R}$ Δ*pomA*Δ*vpsL*	This study
JY369	$vpvC^{W240R}$ Δ*pomA lacZ*:P_{tac} -*mTFP1*:*lacZ*	This study
JY370	$vpvC^{W240R}$ Δ*pomA lacZ*:P_{tac}-*mKate2*:*lacZ*	This study
JY375	$vpvC^{W240R}$ Δ*pomA*Δ*vpsL lacZ*:P_{tac} -*mTFP1*:*lacZ*	This study
JY376	$vpvC^{W240R}$ Δ*pomA*Δ*vpsL lacZ*:P_{tac}-*mKate2*:*lacZ*	This study
Plasmid		
pKAS32	Suicide vector, Amp^R Sm^S	[18]
pNUT144	Suicide vector, Amp^R Kan^R Sm^S	[19]
pNUT157	pNUT144 $vpvC^{W240R}$	[19]
pCMW112	pKAS32 Δ*vpsL*	[20]
pCN003	pKAS32 *lacZ*:P_{tac}-*mTFP1*:*lacZ*	[1]
pCN004	pKAS32 *lacZ*:P_{tac}-*mKate2*:*lacZ*	[1]
pCN005	pKAS32 *lacZ*:P_{tac}-*mKO*:*lacZ*	[19]
pCN007	pKAS32 Δ*rbmA*	[21]
pCN008	pKAS32 Δ*rbmC*	[21]
pCN009	pKAS32 Δ*bap1*	[17]
pCDN010	pKAS32 Δ*pomA*	[21]

Supplementary References

1 Nadell, C. D. & Bassler, B. L. A fitness trade-off between local competition and dispersal in *Vibrio cholerae* biofilms. *Proc. Natl. Acad. Sci. USA* **108**, 14181-14185 (2011).

2 Yildiz, F., Fong, J., Sadovskaya, I., Grard, T. & Vinogradov, E. Structural characterization of the extracellular polysaccharide from *Vibrio cholerae* O1 El-Tor. *PLoS ONE* **9**, e86751 (2014).

3 Phillips, R., Kondev, J., Theriot, J. & Hernan, G. G. *Phyical Biology of the Cell*. 2 edn, (Garland Science, Taylor & Francis Group, 2013).

4 Drescher, K. *et al.* Architectural transitions in *Vibrio cholerae* biofilms at single-cell resolution. *Proc. Natl. Acad. Sci. USA* **113**, E2066-2072 (2016).

5 Kumar, V. S. & Kumaran, V. Voronoi cell volume distribution and configurational entropy of hard-spheres. *J. Chem. Phys.* **123**, 114501 (2005).

6 Xavier, J. B. & Foster, K. R. Cooperation and conflict in microbial biofilms. *Proc. Natl. Acad. Sci. USA* **104**, 876-881 (2007).

7 Woldringh, C. L. & Odijk, T. in *Organization of the Prokaryotic Genome* Ch. 10, (American Society of Microbiology Press, 1999).

8 Güner, A. Unperturbed dimensions and the theta temperature of dextran in aqueous solutions. *J. Appl. Polym. Sci.* **72**, 871-876 (1999).

9 Antoniou, E. & Tsianou, M. Solution properties of dextran in water and in formamide. *J. Appl. Polym. Sci.* **125**, 1681-1692 (2012).

10 Rubinstein, M. & Colby, R. H. *Polymer Physics*. (Oxford University Press, 2003).

11 de Gennes, P.-G. *Scaling Concepts in Polymer Physics*. (Cornell University Press, 1979).

12 Marszalek, P. E., Oberhauser, A. F., Pang, Y.-P. & Fernandez, J. M. Polysaccharide elasticity governed by chair-boat transitions of the glucopyranose ring. *Nature* **396**, 661-664 (1998).

13 Sutherland, I. W. Biofilm exopolysaccharides: a strong and sticky framework. *Microbiology* **147**, 3-9 (2001).

14 Berk, V. *et al.* Molecular architecture and assembly principles of *Vibrio cholerae* biofilms. *Science* **337**, 236-239 (2012).

15 de Lorenzo, V. & Timmis, K. N. Analysis and construction of stable phenotypes in gram-negative bacteria with Tn5- and Tn10-derived minitransposons. *Methods Enzymol.* **235**, 386-405 (1994).

16 Thelin, K. H. & Taylor, R. K. Toxin-coregulated pilus, but not mannose-sensitive hemagglutinin, is required for colonization by *Vibrio cholerae* O1 El Tor and O139 strains. *Infect. Immun.* **64**, 2853-2856 (1996).

17 Yan, J., Sharo, A. G., Stone, H. A., Wingreen, N. S. & Bassler, B. L. *Vibrio cholerae* biofilm growth program and architecture revealed by single-cell live imaging. *Proc. Natl. Acad. Sci. USA* **113**, E5337-E5343 (2016).

18 Skorupski, K. & Taylor, R. K. Positive selection vectors for allelic exchange. *Gene* **169**, 47-52 (1996).

19 Drescher, K., Nadell, C. D., Stone, H. A., Wingreen, N. S. & Bassler, B. L. Solutions to the public goods dilemma in bacterial biofilms. *Curr. Biol.* **24**, 50-55 (2014).

20 Hammer, B. K. & Bassler, B. L. Quorum sensing controls biofilm formation in *Vibrio cholerae*. *Mol. Microbiol.* **50**, 101-104 (2003).

21 Nadell, C. D., Drescher, K., Wingreen, N. S. & Bassler, B. L. Extracellular matrix structure governs invasion resistance in bacterial biofilms. *ISME J.* **9**, 1700-1709 (2015).
